# The Paradox of the Frontal Lobe Paradox. A Scoping Review

**DOI:** 10.3389/fpsyt.2022.913230

**Published:** 2022-07-22

**Authors:** Simon Newstead, Julia Lewis, Gareth Roderique-Davies, Robert M. Heirene, Bev John

**Affiliations:** ^1^Addictions Research Group, School of Psychology and Therapeutic Studies, University of South Wales, Pontypridd, United Kingdom; ^2^Gwent Specialist Substance Misuse Service, Newport, United Kingdom; ^3^School of Psychology, College of Health and Human Sciences, Charles Darwin University, Darwin, NT, Australia

**Keywords:** frontal lobe paradox, knowing-doing dissociation, scoping review, mental capacity, acquired brain injury (ABI)

## Abstract

The “frontal lobe paradox” highlights a phenomenon in which a subset of patients who possess frontal lobe damage and exhibit marked impairments in everyday life are still able to able to verbally describe a logical course of action relating to a task and perform well in interview and test settings. Such cases pose a challenge with regard to the assessment of mental capacity within clinical settings. Recent position articles state that the frontal lobe paradox is a well-known phenomenon within the field of neuropsychology, anecdotal reports from clinicians in the UK suggest this is not the case. Consequently, we conducted a scoping review to examine the breadth and depth of literature relating to the frontal lobe paradox. Searches were conducted using electronic databases and search engines, which were supplemented with a snowball search of the references used within relevant literature. We identified and reviewed 28 documents specifically related to the frontal lobe paradox. Nearly 50% of all identified academic texts published since 2000 were position articles that cited a handful of case studies published between 1936 and 1986 as evidence for the phenomenon. We also observed instances of articles citing position articles as evidence of the frontal lobe paradox. Overall, our findings indicate a lack of readily accessible research specific to the frontal lobe paradox. In particular, there is a lack of contemporary research specific to the subject and an absence of clarification as to which syndromes and disorders are included within the term.

## Introduction

The “frontal lobe paradox” (FLP), otherwise referred to as the “knowing-doing dissociation” (1, 2), highlights a phenomenon in which a subset of people who possess frontal lobe damage and exhibit marked impairments in everyday life are still able to perform well in interview and test settings (3). Individuals are able to verbally describe a logical course of action relating to a task, but then fail to execute it in a real-life scenario (1). Such a dissociation between knowing and doing appears to result from neither a motor deficit or a lack of instructional understanding (4). It may be that such individuals can perform well in office-based tasks due to the clearly defined task rules and requirements, but struggle with ill-structured tasks with no clear rules that are encountered in everyday life (5, 6). One early and well documented example of the FLP describes an accountant, EVR, who underwent bilateral ablation of the orbital and lower medial frontal cortices (7). EVR's ability to organize his life was severely impaired—he was unable to adequately perform his professional responsibilities and function well in day-to day living, with even relatively simple tasks taking hours. Despite this, he retained an IQ of over 130 and had high scores for tests of executive function, such as verbal fluency.

Prefrontal damage related deficits in adaptive and executive functioning can be hard to detect in an interview setting as many of the traditional tests of executive function commonly utilized (e.g., the Wisconsin Card Sorting Test and Stroop test) were not originally intended for use on patient populations (5, 8). In their neuropsychological case series study and review, Burgess et al. (5) concluded that there was an urgent need for an overhaul of clinical assessment procedures to ensure that contemporary neuropsychological testing is based on both experimental findings from cognitive neuroscience and observations of behavior beyond office/clinical environments. Difficulties in assessing functional capacity of individuals with prefrontal damage can be compounded by several factors. These include the preservation of language and verbal reasoning skills (9), allowing such individuals to effectively mask deficits of executive and adaptive functioning (10) and to underestimate their need for support (11). George and Gilbert (10) discuss these issues in relation to the frontal lobe paradox, with relevance to the shortcomings of the UK's Mental Capacity Act (2005) and associated assessment procedures highlighting how a lack of awareness of some aspects of acquired brain injury when assessing capacity may result in professionals deeming patients as much more self-sufficient and competent than they are. This could precipitate a scenario in which individuals who do not possess the mental capacity to appraise their care needs are deemed to be fully capacitous, leaving them vulnerable and without the appropriate support and safeguarding they require (10).

Recent position articles [e.g., (10, 12)] state that the frontal lobe paradox is a well-known phenomenon within the field of neuropsychology, and call for a more efficacious method of assessment with regard to determining mental capacity. However, anecdotal reports from clinicians in the UK suggest that the frontal lobe paradox is not well-known. Further, following these anecdotal reports we briefly scanned the relevant literature and failed to find a substantial body of research on the subject. Consequently, we conducted a full scoping review to examine the breadth and depth of literature relating to the frontal lobe paradox and highlight areas for future research.

## Methods

### Study Design

The aim of our research was to map, report, and discuss the characteristics and concepts within a body of literature, and as such the nature of the research was more suited to scoping review (13) than to a systematic review (14). A scoping review differs from a traditional literature or narrative review in that the review is systematic and often incorporates a comprehensive search for information that is guided by an a priori protocol, therefore including steps to increase reliability and reduce error whilst providing transparency and reproducibility (15). A scoping review typically addresses broader topics where many different study designs might be applicable, without assessing the quality of included studies; whereas a systematic review is more suited to focusing on a well-defined question where appropriate study designs can be identified in advance and the quality of such studies is assessed (16). Additionally, the scoping process incorporates an analytical reinterpretation of the literature, ensuring that data is extracted and presented in a structured way (15, 17, 18).

Our review protocol was based on the scoping review methodological framework proposed by Arksey and O'Malley (16), which employs a five stage process:

1) Identifying the research question
2) Identification of relevant studies
3) Selection of studies/literature
4) Charting the literature and data
5) Collating, summarizing, and reporting the results.

To ensure rigor, we also incorporated several of the recommendations by Levac et al. (18), such as clarifying the purpose of the research and linking this to the research question, balancing breadth and comprehensiveness of the scoping process with what was achievable and feasible, and incorporating both a numerical summary and a qualitative thematic analysis.

### Research Question

The purpose of the present research was to map the relevant literature relating to the frontal lobe paradox. The research question was “What is the available breadth and depth of research and literature on the frontal lobe paradox?”.

### Identification of Relevant Studies

To identify articles and documents relevant to our research question, comprehensive literature searches were conducted between the 01/07/2021 and 23/08/2021 using five electronic databases and search engines. We utilized a six-phrase search string (Table 1) which included the terms “Frontal Lobe Paradox”, “Frontal Lobe Mystery” and “Knowing Doing Dissociation”, all of which are well associated with the FLP. The term “strategy application disorder” was also included in our search as a frontal lobe dysfunction, the etiology of which describes the frontal lobe paradox (19). Similarly, the terms “Frontal lobe Syndrome” and “Dysexecutive Syndrome” were also included in our search as dependent upon the specific behavioral deficits that manifest themselves in real-world situations, individuals may fall under the umbrella of either “frontal” or “dysexecutive” syndromes—two terms which are often used interchangeably but are increasingly becoming distinguished by their behavioral presentation in social situations (20, 21).

**Table 1:** Search protocol.

Search string	“Frontal Lobe Paradox” OR “Frontal Lobe Mystery” OR “Knowing Doing Dissociation” OR “Strategy Application Disorder” OR “Dysexecutive Syndrome” OR “Frontal Lobe Syndrome”
Databases	Medline PsycINFO PubMed Science Direct Scopus
Search engines	Google Google Scholar
Search restrictions	Language: English
Date range	No restrictions

To cast as wide a net as possible in our search for relevant literature, we allowed searches to include the following types of documents and sources of information:

1) Articles in academic journals including case studies, editorials, opinion pieces, studies, and experiments
2) Magazine articles
3) Gray literature (guidance, reports, working papers, government documents, white papers, and evaluations).

To further increase our information capture, snowball searches were conducted on all articles deemed relevant; that is, articles accepted for analysis were searched for additional references relating to our search, which were subsequently sourced and examined. Articles identified by the snowball search but unavailable on either electronic databases or search engines were sourced *via* British Library requests.

### Selection of Studies/Literature

**Figure 1:**
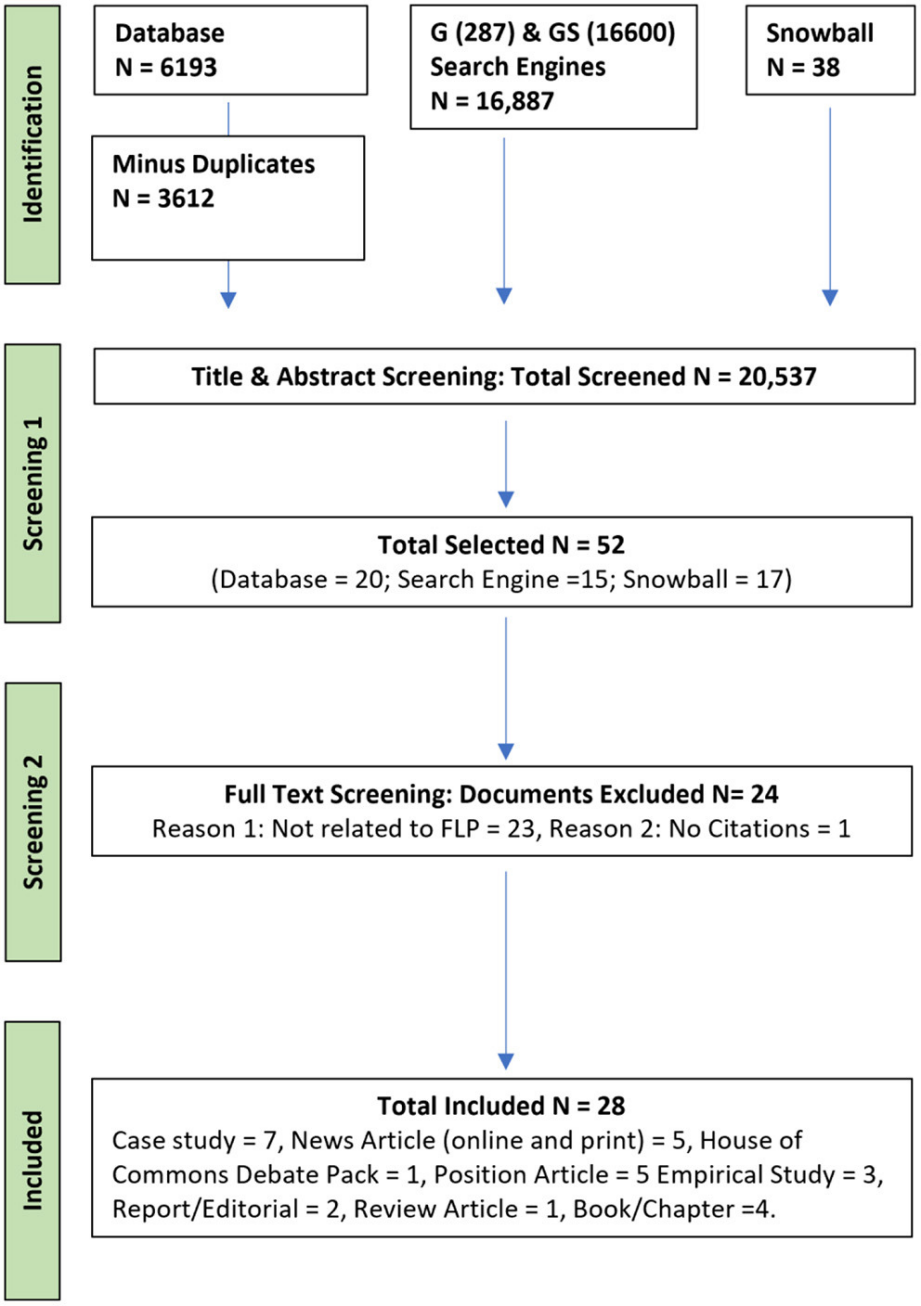
Preferred Reporting Items for Systematic Reviews and Meta-Analyses (PRISMA) flow diagram for scoping review.

Results for search strings were examined in their entirety for searches conducted using electronic databases. The search engine Google initially displays a number of hits that far exceeds the actual number of relevant hits displayed as the additional pages are examined. Therefore, the numbers associated with the Google search (see Figure 1) refer to the relevant hits identified by the search engine.

### Eligibility Criteria and Screening Process

Search results underwent a two-stage screening process. In stage 1, author SN screened titles and abstracts to determine if they held relevance to the nature of our search—that is, they either directly include one of the search phrases and/or directly refer or allude to a dissociation between performance on tests of cognitive function and performance/behavior in real life. In stage 2, SN screened the full text of articles and information sources. A database of all articles identified as potentially relevant during stage 1 screening was created in Excel. Information for articles identified as relevant, during stage 2 screening, was subsequently added to the database. This process was repeated for articles identified during snowball searches.

### Data Charting and Narrative

The data from the articles screened were charted into an Excel database to create a data charting form (https://osf.io/djzut/?view_only=eeafa9f5ec3b4cbcb5efb1676613f72a) which includes a mixture of general information including (where applicable) author, year of publication, item source/location, the type of item, as well as an overview of the information contained within the item. Charting the information from the articles identified in our search enabled us to present both a basic numerical analysis of the various documents included in the review, as well as a narrative of our findings. An inductive approach was taken in the identification of the various themes within the reviewed articles. Articles were manually coded and codes were grouped to produce common themes.

## Results

From the 20,537 results screened at title and abstract level, 52 articles were selected for full-text screening, with 28 articles subsequently identified as relevant to this scoping review. An overview of the identification and screening of articles can be viewed below in Figure 1 and all documents included in the review can be found in Table 2. Consistent with guidance on scoping reviews, we did not appraise the methodological quality of the included articles (15).

**Table 2:** Articles identified by database, search engine, and snowball searches.

**Title**	**Reference**	**Type**	**ID method**
Accepting what we do not know: A need to improve professional understanding of brain Injury in the UK.	(22)	Empirical Study	Snowball
Acquired Brain Injury	(23)	HoC debate pack	Google
Back to Work with a Chronic Dysexecutive Syndrome? (A Case Report)	(24)	Case Study	Database
Behind the cloak of competence: brain injury and mental capacity legislation	(25)	Empirical Study	Database
Decision making and mental capacity: Resolving the Frontal Paradox.	(12)	Position Article	Snowball
Deficits in strategy applications following frontal lobe damage in man	(26)	Case Study	Database
Disorganization of behavior after frontal lobe damage	(27)	Position Article	Database
Frontal cortex and behavior	(28)	Editorial	Snowball
Frontal lobe paradox – how can we best help service users?	(29)	News Article	Google
Frontal lobe paradox and the Mental Capacity Act	(30)	News Article	Google
Frontal lobe paradox: where people have brain damage but don't know it	(31)	News Article	Google
Mental Capacity Act (2005) assessments: why everyone needs to know about the frontal lobe paradox	(10)	Position Article	Google
Mesulam's frontal lobe mystery re-examined	(5)	Review	Database
Parliament and the ‘Frontal Lobe Paradox'	(32)	News Article	Google
Presenting Evidence of Executive Functions Deficit in Court: Why Is Behavior So Important?	(33)	Position Article	Google
Problems assessing executive dysfunction in neurobehavioral disability	(34)	Book Chapter	Snowball
Relationships between measured cognitive ability and reported psychosocial activity after bilateral frontal lobe injury: An 18-year follow-up	(35)	Case Study	GScholar
Report of case of bilateral frontal lobe defect	(36)	Case Study	Snowball
Severe disturbance of higher cognition after bilateral frontal lobe ablation	(7)	Case Study	GScholar
Strategy application disorder: the role of the frontal lobes in human multitasking	(19)	Report	Snowball
The assessment of executive functions: coming out of the office	(37)	Position Article	Snowball
The ecological validity of tests of executive function.	(38)	Empirical Study	Database
The frontal lobe in man: a clinical study of maximum removals	(39)	Case Study	Snowball
The Intellectual Functions Of The Frontal Lobes. (A Study Based Upon Observation Of A Man After Partial Bilateral Lobectomy)	(40)	Book	Snowball
The Riddle of the Frontal Lobe Function in Man	(2)	Book Chapter	Snowball
Understanding Brain Damage: A Primer of Neuropsychological Evaluation	(3)	Book	Snowball
Unilateral frontal lobectomy can produce strategy application disorder	(41)	Case Study	Google
What you need to know: the frontal lobe paradox	(42)	News Article	Google

Table 3 provides an overview of the nine themes identified within the 28 selected texts, subcategorised by document type. Two of the themes fell within our eligibility criteria: describing a discrepancy between neuropsychological tests performance and functional life performance, which was observed in 26 of the documents; and specific mention of the frontal lobe paradox/mystery or knowing-doing dissociation, which was observed in 10 of the documents.

**Table 3:** Themes identified within searched articles.

**Theme**	**Item type**							
	**Case study (7)**	**News article (5)**	**HoC debate pack (1)**	**Position article (5)**	**Empirical study (3)**	**Report/Editorial (2)**	**Review (1)**	**Book/Chapter (4)**	**Total (28)**
Discrepancy between neuropsychological tests performance and functional life performance	7	5	1	5	3	2	1	2	26
Need for changes in practices and testing to enable the identification of the discrepancy/paradox.	0	5	1	4	3	2	1	1	17
Need for changes in practices to increase awareness	0	4	0	3	2	0	0	0	9
Legislation/practice not meeting the needs of individuals with ABI	0	5	1	1	1	0	0	0	8
Inadequacy/poor ecological validity of current tests	0	0	1	4	3	1	1	1	11
Contrasting/testing performance of sufferers of ABI with normal population	0	0	0	0	1	0	0	0	1
Discrepancies between family reports and test results	0	2	1	1	1	0	0	0	5
Importance and role of frontal lobes. Neuroanatomy and function relating to behavior and cognitive assessment	0	0	0	2	0	1	0	2	5
Specifically refers to the frontal lobe paradox/mystery or knowing-doing dissociation.	0	5	1	2	0	0	0	2	10

*() indicates number of each item identified; HoC = House of Commons; ABI = acquired brain injury*.

## Discussion

The aim of this scoping review was to map the relevant literature relating to the frontal lobe paradox, a phenomenon in which a subset of patients exhibit marked impairments in everyday life, but perform well in interview and test settings (3). We identified 28 texts which either directly referred to the frontal lobe paradox/mystery or knowing-doing dissociation, and/or described a dissociation between performance on tests of cognitive function and performance/behavior in real life. Within these examined texts, nine themes that were related to the frontal lobe paradox emerged.

Twenty-two of the documents identified in this review can be classified as academic texts, i.e., published in peer reviewed scientific journals or scientific books. Of these academic texts, 50% of which were published well over 30 years ago. Of these academic texts, 18 were classified as journal articles, which were comprised of five position articles (10, 12, 27, 33, 37), seven case studies (7, 24, 26, 35, 36, 39, 41), one review (5), three empirical studies (22, 25, 38), and two reports/editorials (19, 28). The remainder of academic texts were books or book chapters (2, 3, 34, 40). Notably, six of the seven case studies were documented before the turn of the twenty-first century, and none specifically labeled these cases as an example of either the frontal lobe paradox/mystery or the knowing-doing dissociation. In fact, aside from the texts which coined the phrases (2, 3) the review identified only nine articles which specifically mentioned the frontal lobe paradox/mystery and/or the knowing-doing dissociation, only two of which were academic texts [position articles: (10, 12)]. The other articles which specifically mentioned the frontal lobe paradox included four news articles (29–32) and one House of Commons debate pack (23). Given the supposed familiarity of the FLP within neuropsychology, and recent calls to develop methods of assessment of capacity and executive dysfunction, and calls to raise awareness [e.g., (8, 23, 43)], we had expected to identify a more substantial body of readily available and contemporary research on the subject.

The snowball search of our scoping review was fruitful in identifying texts related to the frontal lobe paradox, with 11 of the 28 texts reviewed located *via* snowball searches. Nine of the texts reviewed were identified via google, two *via* Google Scholar, and just six *via* database searches. Examination of the citations during the snowball search portion of our scoping review highlighted several concerns with regards to the development of a robust body of scientific evidence:

1) Many of the articles in our scoping review which specifically mention the frontal lobe paradox, or the knowing-doing dissociation (which includes all literature disseminated to the general public that we were able to identify) is related to and/or largely predicated on George and Gilberts (10) position article. For example, of the four news articles identified in the review, three were directly linked to George and Gilberts position paper. One which was written by the same authors and published in an online newspaper in the same year (31), essentially provided an overview of the position piece for the general public. Their news article referenced the blog post by the British Psychological Society (32), which itself only cited the position article by (10). The third, a web-based article (42) cited and essentially provided another overview of both George and Gilberts (10) position paper and their news article (31). Additionally, the House of Commons debate pack presented the same information as the blog post by the BPS (32) and only cited the 2018 position article by George and Gilbert.
2) Only nine journal articles were published this century and four of these were position articles. Position articles had a tendency to cite previous position articles, and in some cases the authors of these articles were citing their own work. For example, the position paper by George and Gilbert (10) cited the position paper by Priestley and Manchester (33), which cited a previous position paper they both co-authored (37).
3) Five of the reviewed documents, four of which were commonly cited, were not readily available and had to be requested from the British Library.
4) In general, academic literature associated with the frontal lobe paradox is largely predicated on a very limited, and relatively out-dated body of work. For example, articles often cited as evidence of patients who showed behavioral disorganization in everyday life and the phenomenon of the frontal lobe paradox include Ackerly and Benton (36), Eslinger and Damasio (7), Mesulam (28), Shallice and Burgess (26), and Teuber (2).

The observations made are not an attempt to discredit the work done by the authors of the articles we reviewed, nor insinuate that there is not a need to raise awareness of the frontal lobe paradox and address associated issues such as the implications for mental health capacity assessment. The authors are not disputing that traditional methods for assessing cognitive deficits following frontal lobe damage are not typically capable of measuring the full range of deficits that can occur, nor that the current methods present challenges for clinicians and professionals working with sufferers of ABI (8, 38) and that more efficacious methods are needed for determining mental capacity (12). However, it is important that changes to policy, legislation, and assessment methodology are predicated on a substantial body of reliable scientific evidence, and the observations made within this scoping review certainly raise questions as to whether that is the case. While there is an abundance of research within neuropsychology that attempts to determine the cognitive processes which underpin decision making [e.g., (44–47)], this review highlights an absence of substantial contemporary research specific to the subject of the frontal lobe paradox. While the excellent work by George and Gilbert (10) must be commended for raising awareness of the frontal lobe paradox and its relevance to all professionals involved in assessing mental capacity, the general absence of research specific to the frontal lobe paradox brings with it a real risk that opinions expressed in position articles may increasingly be used as evidence for changes to capacity associated legislation and assessment methodology [e.g., (23)] in place of rigorous empirical research.

## Limitations

The main limitation of the presented research is that the bulk of the scoping review work was carried out by a single individual (author SN). With systematic reviews it is customary to have at least two people independently review the search results, although there are not consistent recommendations regarding the timing of the use of the second reviewer (48). In hindsight, it would have been beneficial to apply this approach to the scoping review as an extra measure of quality control. However, as is customary of a scoping review, no assessment of the quality of included studies was made (16). It may be argued that another limitation of the study is the lack of inclusion of the term “dysexecutive amnesia”, which is a synonym of dysexecutive syndrome.

## Conclusion

This scoping review aimed to map the relevant literature relating to the frontal lobe paradox. Given the broad parameters of the scoping review, the overall number of articles identified in the review (*n* = 28) was limited. This scarcity of literature becomes even more apparent when the types of articles that were identified in the review are examined. One quarter of all academic texts were non-empirical, position articles, which accounted for nearly 50% of all identified academic texts published since the turn of the century. Overall, the findings indicate a lack of readily accessible research specific to the frontal lobe paradox, and in particular a lack of contemporary research specific to the subject. If, as George and Gilbert [(10), p. 56] stated, the frontal lobe paradox is “familiar to many neuropsychologists”, then why does there appear to be such little supporting research specific to the subject? More research specific to the phenomenon of the frontal lobe paradox is needed, and more needs to be done to clarify the syndromes and disorders which may fall under the term. Future research may wish to consider surveying neuropsychologist to try and ascertain the current prevalence of FLP.

## Data Availability Statement

The original contributions presented in the study are included in the article/supplementary materials, further inquiries can be directed to the corresponding author/s.

## Author Contributions

SN, BJ, and GR-D contributed to conception and design of the study. SN conducted the scoping review and organized the database and wrote the first draft of the manuscript. All authors contributed to manuscript revision, read, and approved the submitted version.

## Funding

Funding for open access publication from the University of South Wales, Faculty of Life Sciences and Educations.

## Conflict of Interest

The authors declare that the research was conducted in the absence of any commercial or financial relationships that could be construed as a potential conflict of interest.

## Publisher's Note

All claims expressed in this article are solely those of the authors and do not necessarily represent those of their affiliated organizations, or those of the publisher, the editors and the reviewers. Any product that may be evaluated in this article, or claim that may be made by its manufacturer, is not guaranteed or endorsed by the publisher.
